# The Aging of the AlphaFold Database

**DOI:** 10.1038/s41594-025-01725-z

**Published:** 2025-12-01

**Authors:** Ifigenia Tsitsa, Anja Conev, Alessia David, Suhail A Islam, Michael J E Sternberg

**Affiliations:** 1 Centre for Integrative Systems Biology and Bioinformatics, Department of Life Sciences, Imperial College London, London SW7 2AZ, UK

## Abstract

The AlphaFold database provides 200M protein structures predicted by AlphaFold2 and released in 2022 from sequences in UniProt in April 2021. However, of the 20,504 full-length human structures in the AlphaFold database, 631 entries conflict with the June 2025 release of UniProt (version 2025_03); and there is a similar discrepancy for other species. This highlights how bioinformatics resources, as exemplified by the AlphaFold database can rapidly age.

The AlphaFold database (AlphaFoldDB) ^1^ is a major bioinformatics resource that provides predicted three-dimensional models for over 200 million protein sequences obtained from UniProt ^2^. These models were generated using AlphaFold2 ^3^, the deep learning algorithm developed by DeepMind. The importance of this database is underscored by its subsequent incorporation into UniProt ^2^, one of the most widely used protein databases. These models are extensively used by the community for tasks including molecular replacement (e.g. Phaser ^4^ and MolRep ^5^), missense variant prediction (e.g. FoldX ^6^ and Missense3D ^7^) and as templates in structure prediction (e.g. SwissModel ^8^ and Phyre2.2 ^9^). AlphaFoldDB was based on the protein sequences from April 2021 (UniProtKB version 2021_02, April 2021) which are outdated as UniProt sequences are regularly updated, a discrepancy that researchers should be aware of when studying specific proteins. Moreover, this is a major challenge for developers of general bioinformatics resources that require the maintenance of integrative and robust data management. Here, we have compared AlphaFold models with their respective UniProt sequences across six species and identified these discrepancies. It is vital for the community to be aware of these challenges to preserve the value of such resources.

In August 2025, we performed a comparison of AlphaFoldDB models with the latest UniProt (version 2025_03 i.e. release 3 of 2025 published Jun 18, 2025) for the human proteome. We identified that, among 20,504 unique UniProt accessions with AlphaFold2-predicted structures, 631 sequences differed, corresponding to a 3.08% discrepancy between AlphaFold and UniProt sequences (Figure 1). Out of the conflicting 631 models, 116 correspond to the UniProt sequence entries which were made redundant since the AlphaFoldDB construction based on UniProtKB release 2021_02 ^10^. The redundant UniProt sequences are generally the result of obsolete or reassigned genes, updated taxonomic data, changes in reference databases, annotation errors, or periodic data consolidation to improve consistency and accuracy (*example: Q5TG92*). Sequence length mismatches were observed for 295 models, with UniProt sequences being longer than AlphaFoldDB for 155 models and shorter for 140 models. For example, ZNT2 (Q9BRI3), a zinc transporter protein implicated in the transient neonatal zinc deficiency, has a region of approximately 50 amino acids inserted in the new UniProt sequence around position 100 of the sequence of the outdated AlphaFoldDB model. To assess the impact of this insertion on the ZNT2 structure, we compare model structures (technical details in Supplementary Information). In particular, we compared the AlphaFoldDB model (Figure 1B, blue) with an AlphaFold2 model generated from the updated UniProt sequence (Figure 1B, green). In addition, we regenerated the AlphaFoldDB model using the same sequence and confirmed the low RMSD between the AlphaFoldDB and our regenerated structure (Supplementary Figure 1). We find that the insertion has significant implications for the ZNT2 structure and locations of the transmembrane and zinc binding domains (Figures 1C-D). There were also 221 AlphaFoldDB models where the sequence length was the same, but the sequence differed. In the latter category is Serpin B11 (Q96P1), a non-inhibitory intracellular serpin, with six amino acid substitutions between the two sequences.

To assess the importance of proteins with differing sequences, we found that 406 out of the 631 had high annotation scores (4 and 5), 512 were “reviewed” proteins, and 136 were associated with diseases (as annotated by UniProt). Furthermore, 413 of these proteins were associated with Gene Ontology biological process terms and 418 with molecular function terms, highlighting their potential biological and functional relevance.

We expanded this analysis to include five additional model organisms (Table 1), with mouse showing a very similar trend to human, with 2.5% differing sequences. Interestingly, human and mouse sequences showed greater variation compared to other organisms, probably reflecting the increased depth of research and updates associated with these species. We further investigate this suggestion by looking at the ‘*Date of last sequence modification*’ of UniProt entries across different species. We observe a consistent trend in a higher frequency of changes for human and mouse sequences since 2020 (Supplementary Figure 2). On the other hand, *D. melanogaster* and *A. thaliana* had sequence differences of 0.32% and 0.18% respectively with *S. cerevisiae* showing the least difference between AlphaFoldDB and UniProt sequences from all species (0.08%). Remarkedly *D. rerio* shows a 46.68% difference in its sequences, as nearly half of its proteome is absent from the current UniProt version. This is due to a major curation effort that removed thousands of obsolete or redundant entries, reducing the reviewed *D. rerio* proteome to 3,355 proteins, of which 93 are not present in the AlphaFold database. Notably, some entries, such as Q802X2 and B7ZC96, have AlphaFoldDB models but are excluded from the downloadable version of AlphaFoldDB, likely due to their annotation as “unreviewed” structures. Reviewing their UniProt history reveals they were only recently marked as reviewed (Q802X2 in July 2024 and B7ZC96 in June 2023), well after the last update of AlphaFoldDB in 2022, illustrating both sequence and annotation aging in the AlphaFoldDB. Excluding all the AlphaFoldDB models no longer corresponding to the updated UniProt database, the total number of differing protein sequences is reduced to 10, resulting in a UniProt AlphaFoldDB difference that aligns with trends observed in other organisms.

In conclusion, the AlphaFoldDB provides an unprecedented resource for structural biology but its reliance on outdated sequence and annotation data poses challenges across its widespread applications, including structure-based drug design, variant interpretation and functional analysis. These discrepancies highlight the need for regular updates to maintain alignment with UniProt, especially as other groups, such as SwissModel, continue to develop their own structure databases. One way to mitigate these issues is by using UniProt as the primary reference and mapping sequences to the corresponding AlphaFold models through alignment. We stress the importance of routinely checking consistency between UniProt sequences and AlphaFold models, as underscored by UniProt’s and SWISS-MODEL’s new warning flags and recent community efforts to remodel outdated entries with AlphaSync (https://alphasync.stjude.org/) ^11^. While finalizing this Comment, EMBL-EBI announced release of an updated version of the AlphaFoldDB that corresponds to the latest UniProt release (2025_03). However other resources that build upon earlier deposited AlphaFold models will still be out of date and may not have implemented a warning if their structural model corresponds to an out-of-date UniProt entry. Addressing these issues will be critical for maximising the utility of AlphaFold models and ensuring their integration into downstream tools and resources. Similarly, static bioinformatics databases, including AlphaMissense ^12^, which present precomputed missense variant effect predictions, are also aging. Time flies in bioinformatics.

## Supplementary Material

SI

## Figures and Tables

**Figure 1:**
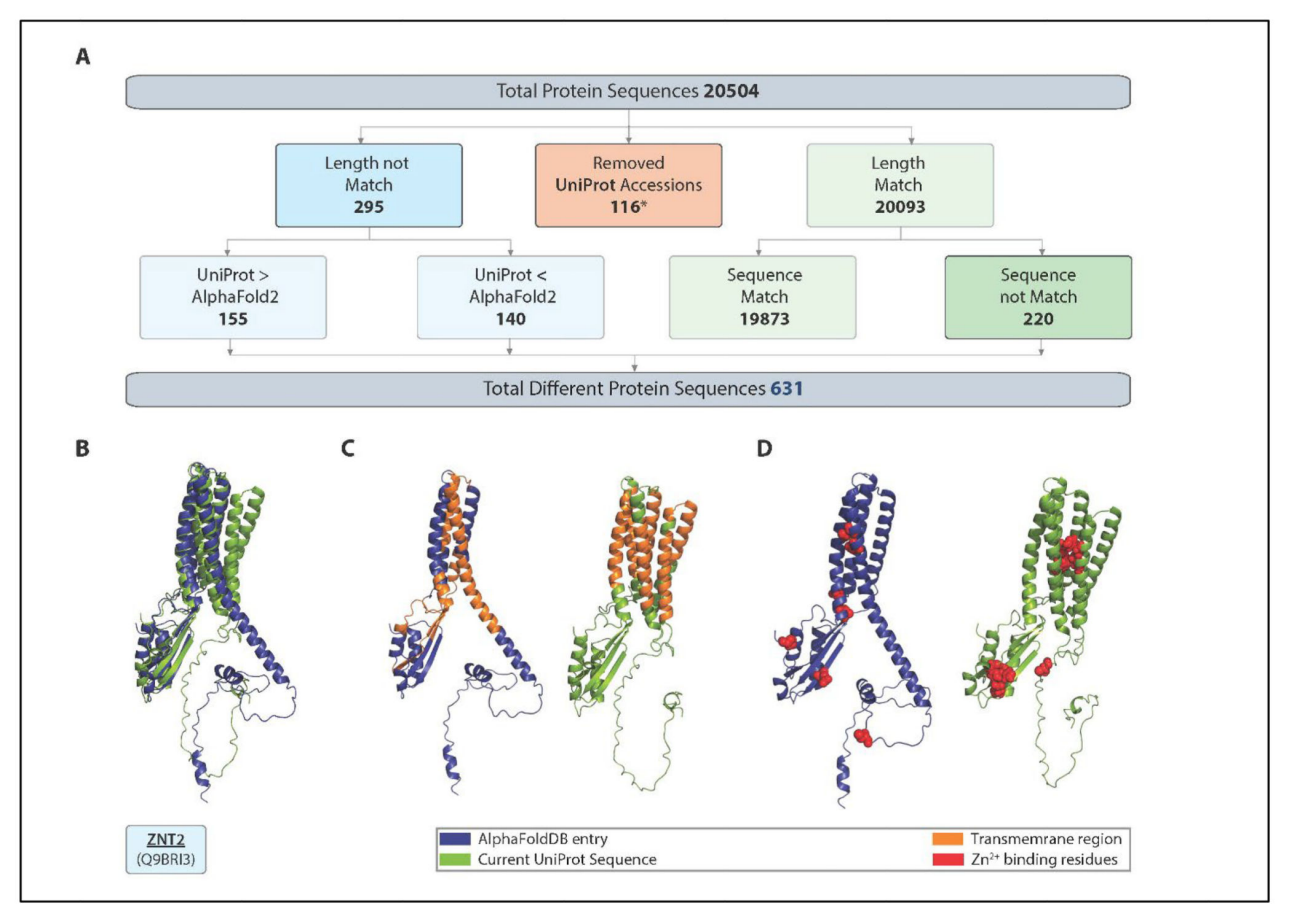
The discrepancy between AlphaFold models built on outdated and current UniProt sequences. (A) Sequence comparison of human AlphaFold2DB models with corresponding UniProt sequences. Blue rectangles indicate cases where the UniProt sequence length has changed, red rectangles mark accessions that have become redundant, and green rectangles denote sequences where UniProt and AlphaFoldDB match in length. (B) Structure models of proton-coupled zinc antiporter ZNT2 (Q9BRI3). The AlphaFold2-predicted structure downloaded from the AlphaFoldDB (blue) is shown aligned with a new AlphaFold2 model generated from the updated UniProt sequence (green). (C) The transmembrane region is highlighted in orange in both the AlphaFoldDB model (blue) and the UniProt-updated model (green), showing the difference in localisation of these regions. (D) Zinc-binding residues are marked in red on both the AlphaFoldDB model (blue) and the UniProt-updated model (green), highlighting their conserved positions in the protein structure. Figures generate using PyMol (The PyMOL Molecular Graphics System, Version 3.1.0 Schrödinger, LLC).

**Table 1:** Summary of the sequence comparison between UniProt and AlphaFoldDB

Species	Common Name	Reference Proteome	Full-length AlphaFoldDB Predicted Structures	Unique UniProt IDs	Total Different Sequences	Percentage of Different Sequences (%)
**H. sapiens**	Human	UP0000056 40	20504	20504	631	3.08
**M. musculus**	Mouse	UP0000005 89	21615	21615	541	2.50
**D.** **melanogast er**	Fruit fly	UP0000008 03	13458	13458	43	0.32
**S.** **cerevisiae**	Budding yeast	UP0000023 11	6039	6039	5	0.08
**D. rerio**	Zebrafish	UP0000004 37	24664	24664	11515	46.68
**A. thaliana**	Arabidopsis	UP0000065 48	27434	27434	50	0.18

## Data Availability

All data used in this analysis is publicly available. The UniProtKB data (release 2025_03) is available at https://ftp.uniprot.org/pub/databases/uniprot/previous_releases/release-2025_03/. The AlphaFoldDB proteomes are available for download at https://ftp.ebi.ac.uk/pub/databases/alphafold/v4/. The analysis is performed using Bash and Python programming languages and the code is available in the Code Ocean capsule: https://codeocean.com/capsule/3156855.
