# Supplementary material for: The Aging of the AlphaFold Database
Source: Nat Struct Mol Biol. Author manuscript; Available in PMC 2026 Feb 14. (PMC7618723; doi:10.1038/s41594-025-01725-z)
Supplement: SI [file EMS211936-supplement-SI.pdf]

The Aging of the AlphaFold Database - Materials and Methods

Sequence Analysis & Comparison

First, AlphaFold models were downloaded from https://alphafold.ebi.ac.uk/download [1] in accordance with Table 1. Next, UniProt sequences for each AlphaFold model were downloaded directly from UniProt [2] along with their respective annotations, including involvement in disease, disruption phenotype, biological process, molecular function Gene Ontology, and date of last sequence modification (version 2025_03, released June 18, 2025). Then, the number of residues in the AlphaFold2/UniProt sequences was compared, and their UniProt Accession Code and Annotation Score was investigated. In addition, the information on the 'Date of last sequence modification' was plotted across different sequences using Python and the Plotly library to highlight the frequency of sequence changes across different sequences. The analysis was performed during August 2025.

AlphaFold2 Model Generation

For the generation of new AlphaFold models, the open-source Colab implementation of AlphaFold2[3] (simplified version 2.3.2) was used. We regenerated the AlphaFoldDB model using the same outdated sequence (Supplementary Figure 1B, lilac). We modelled the updated UniProt sequence (from version 2025_03) of the entry Q9BRI3 (Figure 1B, green). All structures are modelled using the same default parameters of the monomer AlphaFold2 model and include a relaxation run.

Structure feature mapping

The transmembrane regions of Q9BRI3 were mapped using DeepTMHMM [4], and the Zn2+ binding site annotations were extracted from the UniProt feature viewer. All structure visualisations were performed with the PyMol Molecular Graphics System, Version 3.0 Schrödinger, LLC .

Structure model alignment and RMSD calculation

Structures are aligned in PyMol. RMSD is computed using PyMol's rms function. Full atom RMSD between AlphaFoldDB entry and our regenerated AlphaFoldDB model is 15Å. However, when we cut out the long flanking low-pLDDT region (first 65 amino acids of the N-terminus), the resulting full atom RMSD is 0.5Å.

References

1. Varadi, M., Anyango, S., Deshpande, M., Nair, S., Natassia, C., Yordanova, G., Yuan, D., Stroe, O., Wood, G., Laydon, A., et al. (2022). AlphaFold Protein Structure Database: massively expanding the structural coverage of protein-sequence space with high-accuracy models. Nucleic Acids Res. *50*, D439–D444. 10.1093/nar/gkab1061.

2. UniProt Consortium (2023). Uniprot: the universal protein knowledgebase in 2023. Nucleic Acids Res. *51*, D523–D531. 10.1093/nar/gkac1052.

3. Jumper, J., Evans, R., Pritzel, A., Green, T., Figurnov, M., Ronneberger, O., Tunyasuvunakool, K., Bates, R., Žídek, A., Potapenko, A., et al. (2021). Highly accurate protein structure prediction with AlphaFold. Nature *596*, 583–589. 10.1038/s41586-021-03819-2.

4. Hallgren, J., Tsirigos, K.D., Pedersen, M.D., Almagro Armenteros, J.J., Marcatili, P., Nielsen, H., Krogh, A., and Winther, O. (2022). DeepTMHMM predicts alpha and beta transmembrane proteins using deep neural networks. BioRxiv. 10.1101/2022.04.08.487609.

Supplementary Figures

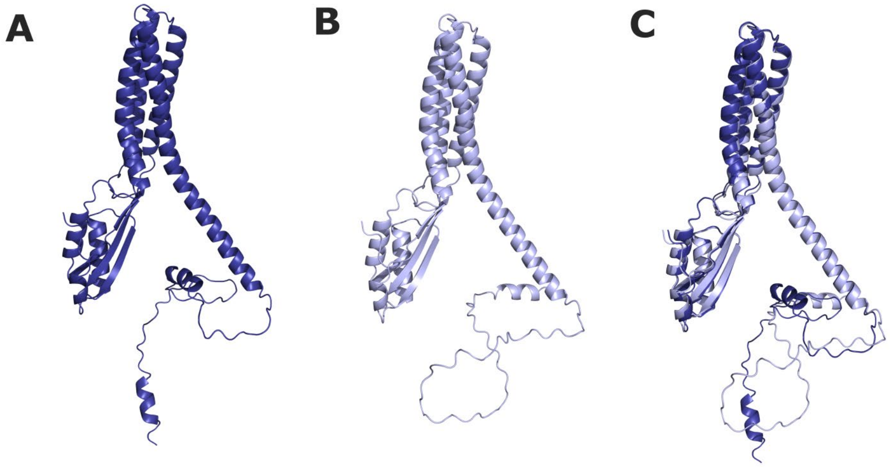

Supplementary Figure 1: Validation of our AlphaFold2 modelling by regenerating the original AlphaFoldDB model. (A) Original AlphaFoldDB entry for Q9BRI3 (blue); (B) Regenerated AlphaFoldDB model for the same outdated Q9BRI3 sequence (lilac); (C) Alignment between the AlphaFoldDB entry and our regenerated AlphaFoldDB model. The models align well overall, except for the loop in the N-terminus low-pLDDT region. The full-atom RMSD is 15Å, while the RMSD excluding the first 65 amino acids of the N-terminus is 0.5Å.

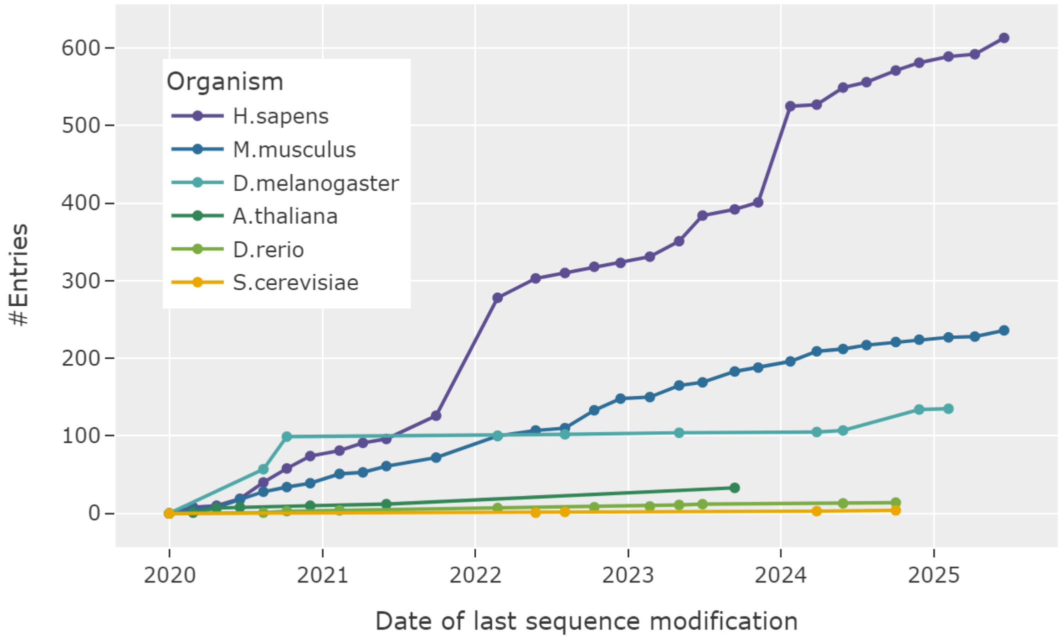

Supplementary Figure 2: Cumulative number of UniProt entries with sequence changes since January 2020 (across species). The x-axis shows the dates of the last sequence change as reported in the UniProt database. In contrast, the y-axis shows the cumulative number of entries that changed between January 2020 and the date indicated on the x-axis. Colours represent different species. Note that 'date of last sequence modification' does not account for the removal of entries from UniProt.
